# Stroke-related mortality analysis in Paraná, Brazil, over 10 years

**DOI:** 10.1055/s-0042-1758398

**Published:** 2022-12-28

**Authors:** Renata Dal-Prá Ducci, Camila Lorenzini Tessaro, Daniela Piera Fontes, Gabriel Schier de Fraga, Raphael Henrique Déa Cirino, Francisco Diego Negrão Lopes Neto, Viviane de Hiroki Flumignan Zetola, Marcos Christiano Lange

**Affiliations:** 1 Universidade Federal do Paraná, Hospital de Clínicas, Divisão de Neurologia, Divisão de Neurologia, Curitiba PR, Brazil.; 2 Universidade Federal do Paraná, Curso de Medicina, Curitiba PR, Brazil.; 3 Faculdades Pequeno Príncipe, Faculdade de Medicina, Curitiba PR, Brazil.; 4 Universidade Federal do Paraná, Hospital de Clínicas, Departamento de Medicina Interna, Divisão de Cardiologia, Curitiba PR, Brazil.; 5 Universidade Federal do Paraná, Hospital de Clínicas, Serviço de Estatística, Curitiba PR, Brazil.

**Keywords:** Stroke, Mortality, Epidemiology, Health Services, Acidente Vascular Cerebral, Mortalidade, Epidemiologia, Serviços de Saúde

## Abstract

**Background** Stroke is the second leading cause of death and disability around the world.

**Objective** The purpose of this study is to evaluate the age- and sex-specific mortality rates related to stroke in the state of Paraná, Brazil, between 2007 and 2016.

**Methods** In this cross-sectional study, residents in the state of Paraná were selected by death certificates (from 2007–2016); the basic cause of death was stroke. A descriptive analysis was performed, and mortality rates were calculated with a 95% confidence interval (95% CI) for each year.

**Results** From 2007 to 2016, there were 62,607 deaths in the state of Paraná due to stroke. Most individuals had medical assistance before death (85.7% in 2007 versus 83.9% in 2016), and most of these deaths occurred in hospitals (73.6% in 2007 versus 74.8% in 2016). Death rates due to stroke increased from 138 (95% CI 135–142) to 163 (95% CI 159–166) per 100,000 inhabitants. This raise occurred mainly in those over 79 years old. For the ages groups of 34 to 44 and 44 to 54 years, mortality rate decreased.

**Conclusions** In the past 15 years, despite the advances in the diagnosis and treatment of stroke, there has been an increase in mortality due to stroke in the state of Paraná. This fact is possibly associated with the aging of the population because there was a more pronounced increase in the group over 79 years old. Thus, new health strategies are necessary to improve the survival and quality of life of poststroke individuals.

## Resumo

**Antecedentes** O acidente vascular cerebral (AVC) é a segunda principal causa de morte e invalidez em todo o mundo.

**Objetivo** O objetivo desse estudo é avaliar as taxas de mortalidade específicas por idade e sexo relacionadas ao AVC no estado do Paraná, Brasil, entre 2007 e 2016.

**Métodos** Neste estudo transversal, foram selecionados residentes no estado do Paraná por certidões de óbito (de 2007–2016); a causa básica da morte foi AVC. Uma análise descritiva foi realizada e as taxas de mortalidade calculadas com intervalo de confiança de 95% (IC 95%) para cada ano.

**Resultados** De 2007 a 2016, ocorreram 62.607 óbitos no estado do Paraná por AVC. A maioria dos indivíduos teve assistência médica antes do óbito (85,7% em 2007 contra 83,9% em 2016), e a maioria desses óbitos ocorreu em hospitais (73,6% em 2007 contra 74,8% em 2016). As taxas de mortalidade por AVC aumentaram de 138 (IC 95% 135–142) para 163 (IC 95% 159–166) por 100.000 habitantes. Esse aumento ocorreu principalmente em maiores de 79 anos. Para as faixas etárias de 34 a 44 e 44 a 54 anos, a taxa de mortalidade diminuiu.

**Conclusões** Nos últimos 15 anos, apesar dos avanços no diagnóstico e tratamento do AVC, houve aumento da mortalidade no estado do Paraná. Este fato está possivelmente associado ao envelhecimento da população, pois houve um aumento mais acentuado no grupo acima de 79 anos. Assim, novas estratégias de saúde são necessárias para melhorar a sobrevida e a qualidade de vida dos indivíduos pós-AVC.

## INTRODUCTION

Stroke is the second leading cause of death and disability worldwide, causing extensive damage to the productive life of individuals. 1 A critical aspect of stroke analysis is that 85% of cases occur in developing countries, where health systems are less effective. 2

From 1990 to 2017, there was a worldwide reduction in stroke incidence, prevalence, mortality, and disability, mainly as a result of primary prevention strategies and advances in the acute management of stroke. 1 3 However, there was an increase in the absolute number of new cases, disability, and deaths. 4 This could be related to the epidemiology and health changes, an increase in life expectancy, and a high frequency of cardiovascular risk factors. 5 It is expected that the aging of the population has increased the incidence of stroke. 6 Besides, 23% of the Global Burden of Disease is attributed to diseases that appear after 60 years of age. In this statistic, neurological causes correspond to almost 7%. Hence, there is an urgent need for public health strategies to adapt to the population aging. There is a higher prevalence of stroke in older people, and health services need to prepare for the increased demand. 7

The aim of the present study is to evaluate age- and sex-specific trends in stroke mortality in the state of Paraná, Brazil, from 2007 to 2016.

## METHODS

In this cross-sectional study, we evaluated the mortality related to stroke, in the state of Paraná, between 2007 and 2016. The manuscript was written according to the strengthening the reporting of observational studies in epidemiology (STROBE) statement for cross-sectional studies (see supplementary material). The present study was approved by the local research ethics committee (Ethics Committee for Research Involving Human Beings of the Paraná State Health Department).

Paraná is the 5 ^th^ largest economy in Brazil and corresponds to 5% of the national population. The state contains almost 200,000 km ^2^ of demographic area with 399 municipalities, and more than 80% of its population lives in urban areas. Besides, its human development index (HDI) in 2010 was 0.749. 8 Curitiba is the capital and the largest city in the state of Paraná; the city's population in 2006 was 1,751,907, 9 and its HDI was 0.823. 8

The number of stroke-related deaths during the study period was obtained from the death register database of the state of Paraná. The stroke deaths were selected by using the international code of diseases (ICD 10) I60 to I69.8. The ICD I67.4 code was not included because it correlates to hypertensive encephalopathy. Individuals who did not present birth data or were not a resident in the state of Paraná were excluded.

The reliability of mortality data in Brazil has increased overtime, and the Southern region of Brazil, which includes the state of Paraná, is historically recognized for the high quality and coverage of mortality data. 10 This is important for reducing study bias.

**Figure 1:**
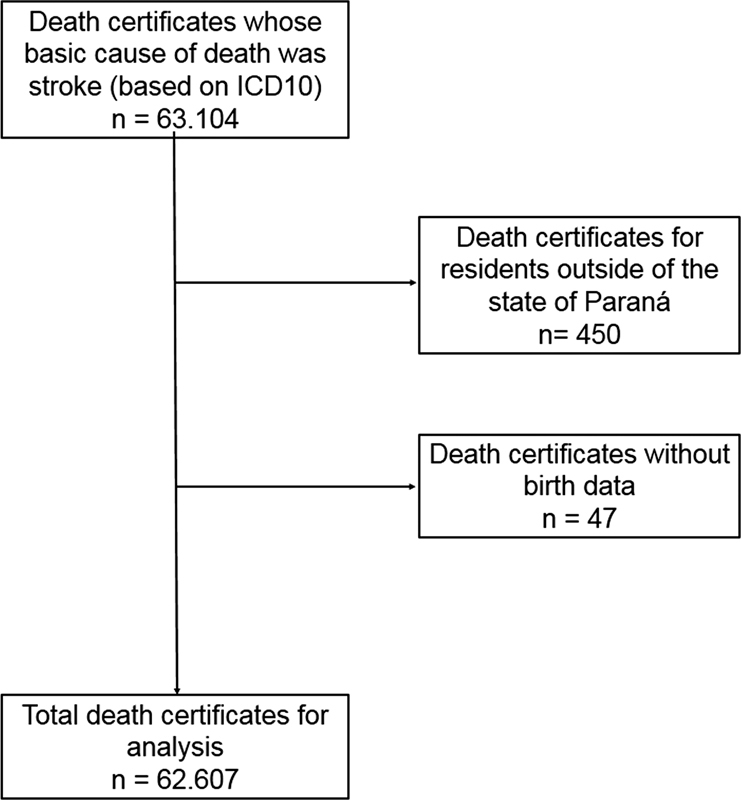
Study methodology flow chart.

Mortality was identified and interpreted based on the death certificates; specifically, the basic (the disease or injury that initiated the events resulting in death), the intermediate (the sequence of events leading to the immediate cause of death), and the immediate (final disease or condition resulting in death) cause of death were evaluated. According to the ICD- 10 and data collected in the medical records, the causes of death were classified as: (1) deaths resulting from any stroke, (2) deaths resulting from ischemic stroke, or (3) deaths resulting from hemorrhagic stroke. For the current study, we considered only patients for whom stroke was the basic cause of death (as described above). Figure 1 shows the criteria applied in the selection of the study individuals.

The following variables were analyzed, according to the information provided by the register database:

- Sex;
- Age;
- Skin color: white, brown, yellow, dark skinned, indigenous;
- Marital status: married, single, divorced, widowed, common-law marriage;
- Schooling: none, 1 to 3 years, 4 to 7 years, 8 to 11 years, 12 or more years;
- Place of death: residence, hospital, others health facilities, other places, public roadway;
- Medical care prior to death: whether the individual received medical care prior to death.

Variables not informed in the register database were grouped into undetermined or ignored.

Quantitative variables were described by mean/average, while qualitative variables were described by frequency and percentage. Stroke mortality rates were analyzed per year and stratified by sex and age using the population midway through the year for each year as the denominator, determined according to the National Population Census. The subgroups according to the age were divided as: 0 to 14, 14 to 24, 24 to 34, 34 to 44, 44 to 54, 54 to 64, 64 to 74, 74 to 79, and > 79 years. Mortality rates were calculated per 100,000 inhabitants during each year of the study period with 95% confidence intervals (CIs), using Soft Page Meta Description.

## RESULTS

From 2007 to 2016, there were 62,607 stroke deaths in the state of Paraná that fulfilled the inclusion criteria of the study. There was a predominance of white (80.1%), male (53.2%), and married (43.2%) individuals.

When comparing 2007 and 2016, there was an increase in the education level: in 2007, 2,468 (41.6%) individuals had 4 or more years of schooling compared with 3,456 (52.7%) in 2016 ( *p* < 0.01). In both years, most individuals had medical assistance before death: 5,082 (85.7%) in 2007 versus 5,498 (83.9%) in 2016 ( *p* = 0.785). Most of these deaths occurred in hospitals: 4,366 (73.6%) in 2007 versus 4,906 (74.8%) in 2016 ( *p* = 0.117). For each year, these and other variables are shown in Table 1 .

**Table 1:** Demographic analysis of stroke death in the state of Paraná during 2007 to 2016

	Year	2007	2008	2009	2010	2011	2012	2013	2014	2015	2016
	Stroke deaths	5933	6250	6204	6377	6298	6223	6238	6260	6268	6556
Sex N (%)	Male	3193 (53.8)	3393 (54.3)	3295 (53.1)	3372 (52.8)	3345 (53.1)	3365 (54.1)	3350 (53.7)	3237 (51.7)	3295 (52.6)	3486 (53.1)
	Female	2740 (46.2)	2857 (45.7)	2909 (46.9)	3005 (47.1)	2953 (46.9)	2858 (45.9)	2888 (46.3)	3023 (48.3)	2973 (47.4)	3070 (46.8)
Mean age		72.13	71.92	72.65	72.79	72.84	73.26	73.57	73.82	73.65	74.09
Skin color N (%)	White	4906 (82.7)	5220 (83.5)	5080 (81.9)	5181 (81.2)	5017 (79.6)	4898 (78.7)	4885 (78.3)	4957 (79.2)	4924 (78.5)	5095 (77.7)
	Brown	592 (9.9)	599 (9.6)	653 (10.5)	719 (11.2)	775 (12.3)	814 (13.1)	836 (13.4)	825 (13.1)	883 (14.1)	967 (14.7)
	Dark skinned	230 (3.9)	219 (3.5)	284 (4.6)	285 (4.4)	317 (5)	306 (4.9)	321 (5.1)	281 (4.5)	294 (4.7)	314 (4.8)
	Yellow	43 (0.7)	52 (0.8)	42 (0.7)	59 (0.9)	44 (0.7)	81 (1.3)	56 (0.9)	65 (1)	75 (1.2)	72 (1.1)
	Indigenous	11 (0.2)	7 (0.1)	5 (0.08)	3 (0.04)	3 (0.04)	9 (0.1)	12 (0.2)	7 (0.1)	5 (0.08)	5 (0.07)
	Undetermined	151 (2.5)	153 (2.4)	140 (2.2)	130 (2)	142 (2.2)	115 (1.8)	128 (2.05)	125 (1.9)	87 (1.4)	103 (1.5)
Marital status N (%)	Married	2762 (46.5)	2822 (45.1)	2800 (45.1)	2827 (44.3)	2769 (43.9)	2658 (42.7)	2631 (42.1)	2516 (40.2)	2604 (41.5)	2665 (40.6)
	Single	644 (10.8)	818 (13.1)	823 (13.2)	814 (12.7)	920 (14.6)	754 (12.1)	765 (12.2)	794 (12.7)	793 (12.6)	882 (13.4)
	Separated/Divorced	272 (4.6)	295 (4.7)	274 (4.4)	294 (4.6)	312 (4.9)	293 (4.7)	339 (5.4)	356 (5.7)	374 (5.9)	403 (6.1)
	Widowed	2066 (34.8)	2167 (34.7)	2192 (35.3)	2333 (36.6)	2188 (34.7)	2148 (34.5)	2189 (35.1)	2284 (36.5)	2199 (35.1)	2271 (34.6)
	Common-law marriage	62 (1)	25 (0.4)	15 (0.2)	6 (0.09)	4 (0.06)	136 (2.2)	130 (2.1)	136 (2.1)	137 (2.2)	154 (2.3)
	Ignored	127 (2.1)	123 (1.9)	100 (1.6)	103 (1.6)	105 (1.6)	234 (3.7)	184 (2.9)	174 (2.7)	161 (2.5)	181 (2.7)
Schooling N (%)	None	1432 (24.1)	1563 (25)	1531 (24.6)	1528 (23.9)	1519 (24.1)	1492 (23.9)	1517 (24.3)	1543 (24.6)	1385 (22.1)	1487 (22.6)
	1–3 years	2033 (34.2)	2187 (34.9)	2252 (36.3)	2167 (33.9)	2231 (35.4)	2043 (32.8)	2078 (33.3)	1960 (31.3)	1827 (29.1)	1613 (24.6)
	4–7 years	1225 (20.6)	1279 (20.4)	1283 (20.7)	1446 (22.6)	1282 (20.3)	1549 (24.9)	1470 (23.5)	1508 (24.1)	1748 (27.9)	2014 (30.7)
	8–11 years	363 (6.1)	451 (7.2)	430 (6.9)	501 (7.8)	511 (8.1)	541 (8.7)	546 (8.7)	549 (8.7)	657 (10.5)	800 (12.2)
	12 or more years	154 (2.6)	185 (2.9)	182 (2.9)	224 (3.5)	243 (3.8)	198 (3.2)	181 (2.9)	207 (3.3)	201 (3.2)	244 (3.7)
	Ignored	726 (12.2)	585 (9.3)	526 (8.4)	511 (8)	512 (8.1)	409 (6.6)	446 (7.1)	493 (7.8)	450 (7.1)	398 (6.1)
Place of death N (%)	Residence	1283 (21.6)	1323 (21.1)	1262 (20.3)	1283 (20.1)	1281 (20.3)	1184 (19)	1183 (18.9)	1133 (18.1)	1192 (19)	1214 (18.5)
	Hospital	4366 (73.6)	4559 (72.9)	4534 (73.1)	4672 (73.2)	4551 (72.2)	4698 (75.5)	4569 (73.2)	4735 (75.6)	4715 (75.2)	4906 (74.8)
	Others health facilities	148 (2.5)	252 (4)	294 (4.7)	317 (4.9)	329 (5.2)	311 (5)	353 (5.6)	286 (4.5)	260 (4.1)	352 (5.3)
	Others	102 (1.7)	78 (1.2)	83 (1.3)	76 (1.2)	101 (1.6)	94 (1.5)	106 (1.7)	83 (1.3)	86 (1.3)	66 (1)
	Public roadway	32 (0.5)	35 (0.5)	31 (0.5)	27 (0.4)	32 (0.5)	24 (0.4)	25 (0.4)	22 (0.3)	14 (0.2)	18 (0.2)
	Ignored	2 (0.03)	3 (0.08)	1 (0.02)	2 (0.03)	4 (0.06)	2 (0.03)	2 (0.03)	1 (0.01)	1 (0.01)	0
Medical care prior to death N (%)	Yes	5082 (85.6)	5345 (85.5)	5322 (85.8)	5321 (83.4)	5285 (83.9)	5135 (82.5)	5274 (84.5)	5254 (83.9)	5299 (84.5)	5498 (83.8)
	No	258 (4.2)	241 (3.8)	293 (4.7)	304 (4.7)	343 (5.4)	284 (4.6)	260 (4.1)	276 (4.4)	277 (4.4)	287 (4.3)
	Ignored	593 (9.9)	664 (10.6)	589 (9.5)	752 (11.8)	660 (10.4)	804 (12.9)	708 (11.3)	730 (11.6)	692 (11)	771 (11.7)

Abbreviation: N (%), absolute number (percentage).

**Figure 2:**
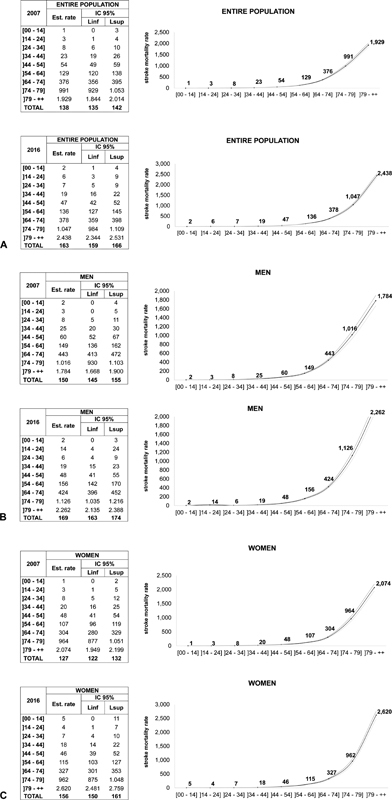
Comparison of stroke mortality rates in 2007 and 2016, stratified by age group, in the state of Paraná, Brazil, for the entire population (A), men (B), and women (C). Abbreviations: CI, confidence interval; Linf, inferior limit; Lsup, superior limit.

During the study period, there was a fluctuation in the number of deaths related to stroke, from 138 (95% CI 135–142), in 2007, to 163 (95% CI 159–166) per 100,000 inhabitants, in 2016 ( Table 2 ). In addition, the age-stratified mortality rate increased from 2007 to 2016, mainly in the > 79-year-old group, in which this difference was statistically significant for men and women ( Figure 2 ). However, for the ages groups of 34 to 44 and 44 to 54 years, the age-stratified mortality rate decreased between the years 2007 and 2016, considering the entire population and also men and women separately ( Figure 2 ).

**Table 2:** Analysis of mortality rates due to stroke in the state of Paraná, Brazil, between 2007 and 2016

Year	∑cases	∑pop (C + F.E + S)	Death rate per 100.000	95%CI	
				Linf	Lsup
**2007**	5,933	4,291,452	138	135	142
**2008**	6,250	4,032,405	155	151	159
**2009**	6,204	4,045,959	153	150	157
**2010**	6,377	4,150,958	154	150	157
**2011**	6,298	4,919,946	161	157	165
**2012**	6,223	4,329,957	144	140	147
**2013**	6,238	4,628,547	172	168	176
**2014**	6,260	4,690,477	170	165	174
**2015**	6,268	4,170,753	150	147	154
**2016**	6,556	4,032,969	163	159	166
**TOTAL**	**62,607**	**40,293,423**	**155**	**154**	**157**

Abbreviations: C, cases; CI, confidence interval; F.E, age group; S,sex; Linf, inferior limit; Lsup, superior limit.

Notes: ∑cases: sum of the cases; ∑pop: sum of population.

**Figure 3:**
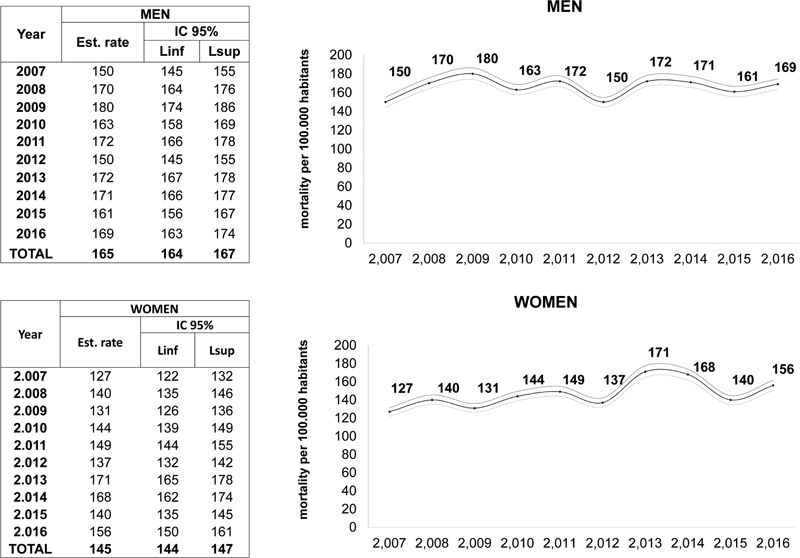
Analysis of stroke mortality rates in men and women in the state of Paraná, Brazil, from 2007 to 2016. Abbreviations: CI, confidence interval; Linf, inferior limit; Lsup, superior limit.

In Paraná, there was a peak of stroke-related mortality between 2013 and 2014, represented by 172 (95% CI 168–176) and 170 deaths (95% CI 165–174) per 100,000 inhabitants, respectively ( Table 2 ). There was a significant increase in the stroke mortality rate for both women and men in those years ( Figure 3 ). The only age range in which women had a higher stroke mortality rate than men was over 79 years ( Figure 2 ).

## DISCUSSION

The present study showed that mortality rates related to stroke oscillated in the state of Paraná from 2007 to 2016. In addition to an increase in the absolute number of deaths, there was an increase in mortality rates, mainly in older individuals, and a decrease in mortality rates among middle-aged individuals. The current global trend is a reduction in mortality rates. 1 In Latin American countries, despite the stroke incidence, prevalence and mortality having followed the global trend, the absolute number of incident strokes increased by 81% from 1990 to 2017, and the absolute numbers of deaths due to stroke increased by 40%. 4

The increase in the mortality rate due to stroke observed in our study, particularly in individuals over 79 years old, can be related to the increase in stroke incidence in elder people, folding every decade after 55 years of age. 11 Stroke deaths increases with age as well, particularly in older individuals (over 65 years old). 12 Furthermore, considering the reduction in fertility rates and mortality and the aging of the population, an increase in stroke incidence and mortality is expected. 13 14 The state of Paraná has followed the accelerated national pattern of population aging, with the last Brazilian Institute of Geography and Statistics Census (2010) indicating 1,316,554 inhabitants over 60 years old in the state, representing 11.2% of the total population. In the 2000 census, the population over 60 years old represented only 8% of the total. 9

Despite the older ages, our study demonstrated a reduction in mortality rates in younger individuals, in the 34 to 44 and 44 to 54 age groups. This is compatible with the data certified by the 2018 *Saúde Brasil* study, conducted by the Ministry of Health, that affirms there was an 11% decrease in stroke mortality in women aged 30 to 69 years from 2010 to 2016. 15 In the same period, there was a decrease from 39.9 to 35.2 deaths per 100,000 inhabitants. Furthermore, there was also a decrease in the incidence of death due to cardiovascular disease from 55 to 51.6 deaths per 100,000 inhabitants. These changes could be related to an improvement in primary care and the use of fewer thrombogenic oral contraceptives, which had been associated with stroke by a women's health study. 16 Another possibility is the reduce in smoking prevalence due to successful government campaigns. 4

Lower financial contributions in public health during this period may also have contributed to the increase in mortality. According to a 2014 study published by the Federal Medical Council, federal, state, and municipal governments invested R$ 3.05 (US$ 0.58) per person per day on health to cover the expenses of the 200 million Brazilian users of the Unified Health System (SUS, in the Portuguese acronym) in 2013. The health expenditure for each citizen in that year was R$ 1,098.75 (US$ 208.9395). 17 This corresponded to 3.6% of the gross domestic product (GDP) invested in health, which is below the international parameter of at least 6%. 18

Between 1979 and 2004, there was an increase in deaths in Brazilian hospitals. Nowadays, more than 5% of those deaths are in consequence of cerebrovascular diseases. 10 These data are similar to the ones found in this study, since there was an increase in hospital deaths during the search. Thus, the increase in the stroke mortality rate may be related to difficulty implementing stroke units in care facilities and to a failure to accomplish secondary prevention and rehabilitation. These data are based on a 5-year cohort study, performed in Paraná, of patients who experienced their first-ever ischemic stroke in the middle cerebral artery territory: 45.3% of poststroke deaths occurred due to infections, mainly pneumonia (79.1%), and not due to cardiovascular diseases as in other studies. These findings suggest a failure in rehabilitation capacity and quality as well as in the long-term follow-up of these patients. 19

In our study, in the age group > 79 years, the stroke mortality rate was significantly higher in women compared with men. A 2017 study including 60 Brazilian cities reported that more than 50% of deaths due to stroke were in women. 20 Higher stroke mortality in women is probably due to greater severity of stroke, higher prevalence of atrial fibrillation and functional limitations, and greater longevity of women compared with men. 21 22

Some limitations regarding the present study should be considered. The data were obtained retrospectively through analysis of death certificates. This fact limited the quality of analysis of some variables, such as schooling and health care. When comparing our data with those of the people living in Paraná in the same period, a divergence is noted in the variables of sex, schooling, and marital status. In the Paraná general population analysis, there is no sex predominance; although most people have no schooling or only 1 to 3 years of schooling (48.7%), there is a smaller proportion of individuals who have 4 to 7 years of schooling (18.1%) and a higher proportion of those who have 8 to 11 years (22.9%) and 12 years or more (9.7%); the majority of the population of Paraná is single (47.9%), followed by the married individuals (44.2%), with a smaller proportion of widowers (5.4%). Similar to our study, there is a predominance of white individuals (70.3%). The variable “access to medical care” was not analyzed for people living in Paraná, as it is a databased on death certificates. 9

Nevertheless, information obtained through death certificates is considered reliable, 10 and Brazilian mortality data are viewed as accurate and reliable for epidemiological analysis. 23 Moreover, it was not possible to distinguish the mortality rates due to ischemic or hemorrhage stroke because the stroke subtype was not indicated in most of the death certificates.

The importance of this study is due to its originality and relevance, mainly because it shows an epidemiological study about stroke-related mortality over a period of 10 years in Brazil. In addition, the results of the study will be important as comparative data to analyze the impact of the COVID-19 pandemic on the care of patients with stroke.

In the past 15 years, despite the advances in the diagnosis and treatment, there was an increase in stroke mortality in the state of Paraná. This fact is possibly related to the aging of the population, since there was a more pronounced increase in the group over 79 years old. The implementation of actions and policies to prevent and treat stroke, both individually and collectively, are fundamental. For that reason, there should be an incentive for the State Integrated Stroke Care Line to improve care both during the acute phase of stroke and after individuals are discharged from the hospital.

## References

[1] GBD 2016 Stroke Collaborators Global, regional, and national burden of stroke, 1990-2016: a systematic analysis for the Global Burden of Disease Study 2016 Lancet Neurol 2019 18 05 439 458 30871944 10.1016/S1474-4422(19)30034-1 PMC6494974

[2] Donnan G A Stroke around the world Int J Stroke 2011 6 03 181 21557800 10.1111/j.1747-4949.2011.00600.x

[3] Thrift A G Thayabaranathan T Howard G Global stroke statistics Int J Stroke 2017 12 01 13 32 27794138 10.1177/1747493016676285

[4] Ouriques Martins S C Sacks C Hacke W Priorities to reduce the burden of stroke in Latin American countries Lancet Neurol 2019 18 07 674 683 31029579 10.1016/S1474-4422(19)30068-7

[5] Borges G M Health transition in Brazil: regional variations and divergence/convergence in mortality Cad Saude Publica 2017 33 08 e00080316 28832781 10.1590/0102-311X00080316

[6] Feigin V L Vos T Global Burden of Neurological Disorders: From Global Burden of Disease Estimates to Actions Neuroepidemiology 2019 52(1-2):1 2 30472717 10.1159/000495197

[7] Murray C JL Lopez A D Measuring global health: motivation and evolution of the Global Burden of Disease Study Lancet 2017 390(10100):1460 1464 28919120 10.1016/S0140-6736(17)32367-X

[8] Atlas do Desenvolvimento Humano do Brasil Ranking Todo o Brasil, www.atlasbrasil.org.br/ranking 2013, accessed 13 July 2021).

[9] IBGE Instituto Brasileiro de Geografia e Estatística. Censo 2010 https://censo2010.ibge.gov.br/(Accessed 13 July 2021).

[10] Departamento de Informática do SUS Informações em saúde, www2.datasus.gov.br/DATASUS/(2009, accessed 5 November 2020].

[11] Ovbiagele B Nguyen-Huynh M N Stroke epidemiology: advancing our understanding of disease mechanism and therapy Neurotherapeutics 2011 8 03 319 329 21691873 10.1007/s13311-011-0053-1 PMC3250269

[12] Yousufuddin M Young N Aging and ischemic stroke Aging (Albany NY) 2019 11 09 2542 2544 31043575 10.18632/aging.101931 PMC6535078

[13] Nasri F The aging population in Brazil Einstein (Sao Paulo) 2008 6 01 S4 S6

[14] Donkor E S Stroke in the 21 ^st^ Century: A Snapshot of the Burden, Epidemiology, and Quality of Life Stroke Res Treat 2018 2018 3.238165E6 10.1155/2018/3238165 PMC6288566 30598741

[15] Ministério da Saúde do Brasil Saúde Brasil 2018 - Uma análise da situação de saúde e das doenças e agravos cronicos: desafios e perspectivas, http://bvsms.saude.gov.br/bvs/publicacoes/saude_brasil_2018_analise_situacao_saude_doencas_agravos_cronicos_desafios_perspectivas.pdf(2019, accessed 23 October 2020).

[16] Wassertheil-Smoller S Kaplan R C Salazar C R Stroke findings in the Women's Health Initiative Semin Reprod Med 2014 32 06 438 446 25321421 10.1055/s-0034-1384627

[17] Júnior M Governo gasta R$ 3,89 ao dia na saúde de cada brasileiro, https://portal.cfm.org.br/index.php?option=com_content&id=25985:2016-02-18-12-31-38(2019, accessed 09 September 2020).

[18] World Health Organization (Regional Committee for the Americas), Strategy for Universal Access to Health and Universal Health Coverage 2014

[19] Ducci R D Lange M C Zétola V HF Rundek T Factors Related to Cardioembolism as Major Predictors of Poor Survival after First-Ever Middle Cerebral Artery Stroke Treated with Thrombolysis Cerebrovasc Dis 2017 43(3-4):178 185 28208130 10.1159/000455723

[20] Mamed S N Ramos A MO Araújo V EM Jesus W S Ishitani L H França E B Profile of deaths from unspecified stroke after investigation of garbage codes in 60 cities in Brazil, 2017 Rev Bras Epidemiol 2019 22(22, Suppl 3)e190013, 3 31800852 10.1590/1980-549720190013.supl.3

[21] Phan H T Blizzard C L Reeves M J Sex Differences in Long-Term Mortality After Stroke in the INSTRUCT (INternational STRoke oUtComes sTudy): A Meta-Analysis of Individual Participant Data Circ Cardiovasc Qual Outcomes 2017 10 02 e003436 28228454 10.1161/CIRCOUTCOMES.116.003436

[22] Phan H T Gall S Blizzard C L Sex Differences in Causes of Death After Stroke: Evidence from a National, Prospective Registry J Womens Health (Larchmt) 2021 30 03 314 323 33227218 10.1089/jwh.2020.8391

[23] Laurenti R Mello Jorge M HPD Gotlieb S LD A confiabilidade dos dados de mortalidade e morbidade por doenças crônicas não transmissíveis Cien Saude Colet 2004 9 04 909 920

